# Gene model for the ortholog of *Ilp5* in *Drosophila ananassae*

**DOI:** 10.17912/micropub.biology.000782

**Published:** 2024-12-08

**Authors:** Megan E. Lawson, Madeline McAbee, Rae A. Lucas, Scott Tanner, Jacqueline Wittke-Thompson, Tara A. Pelletier, Zeynep Ozsoy, Rachel Sterne-Marr, Chinmay P. Rele, Laura K Reed

**Affiliations:** 1 University of Alabama, Tuscaloosa, Alabama, US; 2 University of South Carolina Upstate, Spartanburg, SC USA; 3 University of St. Francis, Joliet, IL USA; 4 Radford University, Radford, VA USA; 5 Colorado Mesa University, Grand Junction, CO, USA; 6 Siena College, Loudonville, NY USA

## Abstract

Gene model for the ortholog of Insulin-like peptide 5 ( *Ilp5* ) in the *D. ananassae* May 2011 (Agencourt dana_caf1/DanaCAF1) Genome Assembly (GenBank Accession: GCA_000005115.1 ) of *Drosophila ananassae* . This ortholog was characterized as part of a developing dataset to study the evolution of the Insulin/insulin-like growth factor signaling pathway (IIS) across the genus *Drosophila* using the Genomics Education Partnership gene annotation protocol for Course-based Undergraduate Research Experiences.

**Figure 1. Genomic neighborhood and gene model for Ilp5 in D. ananassae:**
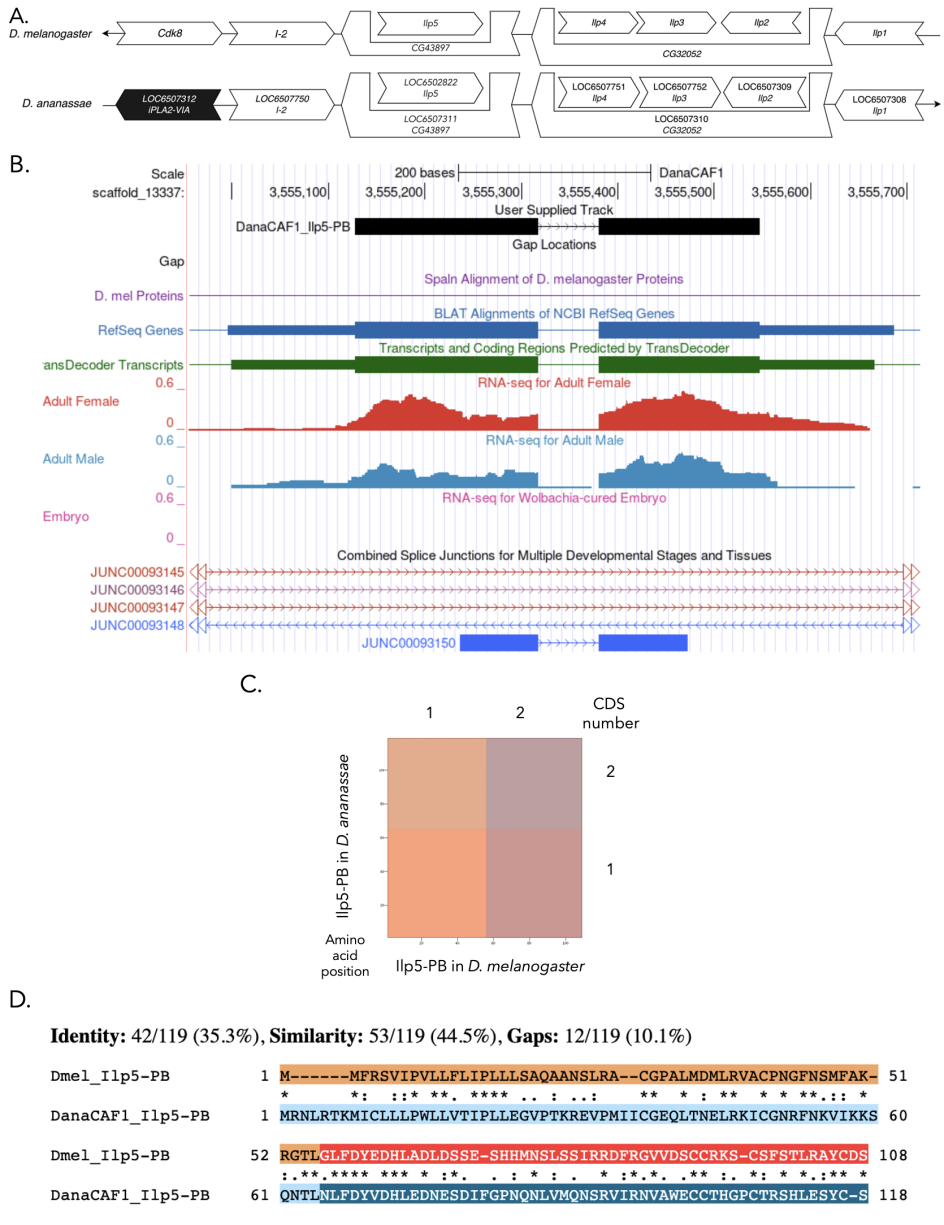
(A) Synteny of genomic neighborhood of *Ilp5* in *D. melanogaster* and *D. ananassae* . Gene arrows pointing in the same direction as the target gene in both *D. ananassae* and *D. melanogaster* are on the same strand as the target gene; gene arrows pointing in the opposite direction are on the opposite strand. The thin underlying arrows pointing to the right indicate that *Ilp5* is on the + strand; arrows pointing to the left indicate that *Ilp5* is on the – strand. White arrows in *D. ananassae* indicate the locus ID and orthology to the corresponding gene in *D. melanogaster* , and black arrows indicate a non-orthologous gene. The gene names given in the *D. ananassae* gene arrows indicate the orthologous gene in *D. melanogaster* , while the locus identifiers are specific to *D. ananassae* . (B) Gene Model in UCSC Track Hub (Raney et al. 2014): the gene model in *D. ananassae* (black), Spaln of *D. melanogaster* Proteins (purple, alignment of RefSeq proteins from *D. melanogaster* ), BLAT alignments of NCBI RefSeq Genes (blue, alignment of RefSeq genes for *D. ananassae* ), RNA-Seq from adult females (red), adult males (blue), and Wolbachia-cured embryos (pink) (alignment of Illumina RNA-Seq reads from *D. ananassae* ), and Transcripts (green) including coding regions predicted by TransDecoder and Splice Junctions Predicted by regtools using *D. ananassae* RNA-Seq (Gravely et al., 2011; SRP006203, PRJNA257286, SRP007906, PRJNA388952). The splice junction supporting the Ilp5-PB model has a read-depth of 30, with 10-49, 100-499, and >1000 supporting reads in blue, pink, and red respectively. The custom gene model (User Supplied Track) is indicated in black with CDSs depicted by boxes and introns by narrow lines (arrows indicate direction of transcription). (C) Dot Plot of Ilp5-PB in *D. melanogaster* ( *x* -axis) vs. the orthologous peptide in *D. ananassae* ( *y* -axis). Amino acid number is indicated along the left and bottom; CDS number is indicated along the top and right; CDSs are also highlighted with alternating colors. The default word size required to indicate sequence similarity in this dot plot is six, and as there are not any completely conserved sequences that span larger than six amino acids in this gene, no line is shown. (D) Protein alignment of Ilp5-PB in *D. melanogaster* (top row) vs. the orthologous peptide in *D. ananassae* (bottom row). The amino acids in Ilp5-PB are indicated in each species' respective row, with CDSs being highlighted in alternating colors. Asterisks between the protein alignment from each species indicate that the amino acid at that position is the same, colons and periods indicate the amino acids are very similar structurally (colons at a higher extent and periods at a lesser extent), and empty space indicates the amino acids are not similar. Lines in the protein sequence in either species indicate that there are gaps in that portion of the alignment for that species.

## Description

**Table:** 

*This article reports a predicted gene model generated by undergraduate work using a structured gene model annotation protocol defined by the Genomics Education Partnership (GEP; thegep.org) for Course-based Undergraduate Research Experience (CURE). The following information in this box may be repeated in other articles submitted by participants using the same GEP CURE protocol for annotating Drosophila species orthologs of Drosophila melanogaster genes in the insulin signaling pathway.* "In this GEP CURE protocol students use web-based tools to manually annotate genes in non-model *Drosophila* species based on orthology to genes in the well-annotated model organism fruitfly *Drosophila melanogaster* . The GEP uses web-based tools to allow undergraduates to participate in course-based research by generating manual annotations of genes in non-model species (Rele et al., 2023) . Computational-based gene predictions in any organism are often improved by careful manual annotation and curation, allowing for more accurate analyses of gene and genome evolution (Mudge and Harrow 2016; Tello-Ruiz et al., 2019) . These models of orthologous genes across species, such as the one presented here, then provide a reliable basis for further evolutionary genomic analyses when made available to the scientific community.” (Myers et al., 2024) . “The particular gene ortholog described here was characterized as part of a developing dataset to study the evolution of the Insulin/insulin-like growth factor signaling pathway (IIS) across the genus *Drosophila* . The Insulin/insulin-like growth factor signaling pathway (IIS) is a highly conserved signaling pathway in animals and is central to mediating organismal responses to nutrients (Hietakangas and Cohen 2009; Grewal 2009) .” (Myers et al., 2024) .

We propose a gene model for the *D. ananassae* ortholog of the *D. melanogaster* Insulin-like peptide 5 ( *Ilp5* ) gene. The genomic region of the ortholog corresponds to the uncharacterized protein XP_001956270.2 (Locus ID LOC6502822 ) in the May 2011 (Agencourt dana_caf1/DanaCAF1) Genome Assembly of *D. ananassae* ( GCA_000005115.1 - Drosophila 12 Genomes Consortium et al., 2007). This model is based on RNA-Seq data from *D. ananassae* ( SRP006203 , PRJNA257286 , SRP007906 , PRJNA388952 ) and *Ilp5* in *D. melanogaster* using FlyBase release FB2023_03 ( GCA_000001215.4 ; Larkin et al., 2021; Gramates et al., 2022; Jenkins et al., 2022).

In animals, the pathway plays a central role in growth, development, reproduction, in addition to metabolism (Semaniuk et al., 2021) . In *Drosophila* , signaling starts when Insulin-like peptides (Ilp), of which there are eight, bind to their receptors. Ilp5 shows particular conservation from *Drosophila* to mammals, binding to insect insulin-binding proteins, and also shown to lower blood glucose levels when administered to rats (Sajid et al., 2011) . In *Drosophila* , *Ilp5* expression is highest in the insulin-producing cells in the brain, but also found expressed in the gut and ovaries of larvae, and neuroendocrine cells of both adults and larva (Semaniuk et al., 2021) . Flies lacking Ilp5 function show defects in fertility, (Grönke et al. 2010; Strilbystka et al. 2020) , behavior (Semaniuk et al. 2018; Strilbystka et al. 2020) , metabolism (Semaniuk et al. 2018; Strilbystka et al. 2020) , and lifespan (Grönke et al. 2010) .

*D* . *ananassae* (NCBI:txid7217) is part of the *melanogaster* species group within the subgenus *Sophophora* of the genus *Drosophila* (Sturtevant 1939; Bock and Wheeler 1972) . It was first described by Doleschall (1858). *D. ananassae* is circumtropical (Markow and O’Grady 2005; https://www.taxodros.uzh.ch, accessed 1 Feb 2023), and often associated with human settlement (Singh 2010) . It has been extensively studied as a model for its cytogenetic and genetic characteristics, and in experimental evolution (Kikkawa 1938; Singh and Yadav 2015) .

***Synteny***

*Ilp5* occurs on chromosome 3L in *D. melanogaster* and is nested by *CG43897 ,* flanked upstream by *I-2* and *Cdk8 ,* and flanked downstream by *CG32052* and *Ilp1 . CG32052* nests *Ilp4 , Ilp3 ,* and *Ilp2 .* We determined that the putative ortholog of *Ilp5* is found on scaffold 13337 ( CH902618.1 ) in *D. ananassae* (CAF1 assembly GCA_000005115.1 ) with LOC6502822 ( XP_001956270.2 ) (via *blastp* search with an e-value of 8e-09 and percent identity of 39.51%), where it is nested by LOC6507311 ( XP_032310388.1 ), which corresponds to *CG43897* in *D. melanogaster* with an e-value of 0.0 and percent identity of 68.77%, as determined by *blastp* . The *Ilp5* ortholog is flanked upstream by LOC6507750 ( XP_001956268.3 ) and LOC6507312 ( XP_001956267.1 ), which correspond to *I-2* and *iPLA2-VIA* in *D. melanogaster* with e-values of 1e-84 and 0.0 respectively and percent identities of 72.50% and 90.87% respectively, as determined by *blastp* . The *Ilp5* ortholog is flanked downstream by LOC6507310 ( XP_014765147.1 ) and LOC6507308 ( XP_001956275.2 ), which correspond to *CG32052* and *Ilp1* in *D. melanogaster* with e-values of 0.0 and 4e-24 respectively and percent identities of 86.67% and 52.46% respectively, as determined by *blastp* . The *CG32052* ortholog ( LOC6507310 , XP_014765147.1 ) nests LOC6507751 ( XP_032309882.1 ), LOC6507752 ( XP_001956273.1 ), and LOC6507309 ( XP_001956274.1 ), which correspond to *Ilp4 , Ilp3 ,* and *Ilp2* in *D. melanogaster* with e-values of 7e-28, 1e-21, and 2e-27 respectively and percent identities of 48.91%, 46.32%, and 46.79% respectively, as determined by *blastp* ( Figure 1A, A ltschul et al., 1990). We argue this is the correct ortholog assignment for *Ilp5* in *D. ananassae* because all but one gene in the local genomic neighborhood are syntenic and all of the *blastp* results used to determine orthology were of high-quality.

***Protein Model***

*Ilp5* in *D. ananassae* has one protein coding isoform, Ilp5-PB ( Figure 1B ). Isoform Ilp5-PB contains two CDSs. This is the same relative to the ortholog in *D. melanogaster* , which also has one protein-coding isoform with two CDSs. The sequence of *Ilp5* in *D. ananassae* has 35.3% identity with the *Ilp5* in *D. melanogaster* as determined by *blastp* ( Figure 1C ). The coordinates of the curated gene models can be found in NCBI at GenBank/BankIt using the accession BK064649. These data are also available in Extended Data files below, which are archived in CaltechData.

***Special characteristics of the Protein Model***

The dot plot for the alignment of the Ilp5-PB sequences in *D. melanogaster* and *D. ananassae* (Figure C) does not show any regions of sequence similarity. This is due to the default parameters of the dot plot requiring a word size of six, which is not present. However, the protein alignment (Figure D) shows that while there are no consecutive identities of six or higher, there are regions of Ilp5-PB that are conserved between *D. ananassae* and *D. melanogaster.* The lack of a full six-length word could be the result of mutations accumulating over time, in combination with the small size of this gene.

## Methods

Detailed methods including algorithms, database versions, and citations for the complete annotation process can be found in Rele et al. (2023). Briefly, students use the GEP instance of the UCSC Genome Browser v.435 ( https://gander.wustl.edu; Kent WJ et al., 2002; Navarro Gonzalez et al., 2021) to examine the genomic neighborhood of their reference IIS gene in the *D. melanogaster* genome assembly (Aug. 2014; BDGP Release 6 + ISO1 MT/dm6). Students then retrieve the protein sequence for the *D. melanogaster* target gene for a given isoform and run it using *tblastn* against their target *Drosophila* species genome assembly ( *D. ananassae;* GCA_000005115.1 ) on the NCBI BLAST server (https://blast.ncbi.nlm.nih.gov/Blast.cgi, Altschul et al., 1990) to identify potential orthologs. To validate the potential ortholog, students compare the local genomic neighborhood of their potential ortholog with the genomic neighborhood of their reference gene in *D. melanogaster* . This local synteny analysis includes at minimum the two upstream and downstream genes relative to their putative ortholog. They also explore other sets of genomic evidence using multiple alignment tracks in the Genome Browser, including BLAT alignments of RefSeq Genes, Spaln alignment of D. melanogaster proteins, multiple gene prediction tracks (e.g., GeMoMa, Geneid, Augustus), and modENCODE RNA-Seq from the target species. Genomic structure information (e.g., CDSs, CDS boundaries, number of isoforms) for the *D. melanogaster* reference gene is retrieved through the Gene Record Finder (https://gander.wustl.edu/~wilson/dmelgenerecord/index.html; Rele et al *.,* 2023). Approximate splice sites within the target gene are determined using *tblastn* using the CDSs from the *D. melanogaste* r reference gene. Coordinates of CDSs are then refined by examining aligned modENCODE RNA-Seq data, and by applying paradigms of molecular biology such as identifying canonical splice site sequences and ensuring the maintenance of an open reading frame across hypothesized splice sites. Students then confirm the biological validity of their target gene model using the Gene Model Checker (https://gander.wustl.edu/~wilson/dmelgenerecord/index.html; Rele et al., 2023), which compares the structure and translated sequence from their hypothesized target gene model against the *D. melanogaster* reference gene model. At least two independent models for each gene are generated by students under mentorship of their faculty course instructors. These models are then reconciled by a third independent researcher mentored by the project leaders to produce a final model like the one presented here. Note: comparison of 5' and 3' UTR sequence information is not included in this GEP CURE protocol.

## Extended Data

Description: ZIP file containing the FNA, FAA, and GTF of the generated model.. Resource Type: Model. DOI: 10.22002/56zzc-nhb19
